# An update on the pathogenesis of Hashimoto’s thyroiditis

**DOI:** 10.1007/s40618-020-01477-1

**Published:** 2020-12-17

**Authors:** A. P. Weetman

**Affiliations:** Department of Oncology and Metabolism, Faculty of Medicine, Dentistry and Health, University of Sheffield, The Medical School, Beech Hill Road, Sheffield, S10 2RX UK

**Keywords:** Hashimoto’s thyroiditis, Autoimmunity, Thyroid antibodies

## Abstract

It is 70 years since Noel Rose embarked on his pioneering studies that lead to the discovery of autoimmune thyroiditis and the elucidation of Hashimoto’s thyroiditis. This short review to honour his passing focuses on the developments in our understanding of the causes and pathogenesis of HT over the last five years. Recent genetic studies have reported heritability estimates for HT and associated diseases for the first time, and emphasised the complexity of the genetic factors involved, including monogenic forms of HT. Environmental factors continue to be elucidated, especially as a side effect of drugs which modulate the immune system therapeutically. Regarding pathogenetic mechanisms, multiple cytokine networks have been identified which involve the thyroid cells in a circuit of escalating proinflammatory effects, such as the expression of inflammasome components, and an array of different defects in T regulatory cells may underlie the loss of self-tolerance to thyroid autoantigens. Finally, a number of studies have revealed fresh insights into disease associations with HT which may have both pathological and clinical significance, the most intriguing of which is a possible direct role of the autoimmune process itself in causing some of the persistent symptoms reported by a minority of patients with levothyroxine-treated HT.

## Introduction

Seventy years ago, Noel Rose began his medical school studies at the University of Buffalo, joining the laboratory of Ernest Witebsky where he was given the task of preparing pure thyroglobulin. Rose tested the resulting preparations for any degree of denaturation by seeing if they would provoke an immune response when injected into rabbits. The then current dogma held that the body could not recognise self-proteins due to the teleological phenomenon of ‘*horror autotoxicus*’ so any immune response would imply the protein was degraded and no longer ‘self.’ Although there was indeed no response following intravenous injection, subsequent experiments using an adjuvant to stimulate the immune system and injection into the animal’s footpad led to the entirely unexpected formation of thyroglobulin antibodies. His supervisor initially doubted the finding and made Rose repeat it many times. Eventually Rose purified thyroglobulin which was injected back into the very same animal from which it was derived and found not only antibodies but also inflammation in the thyroid: he had discovered autoimmune thyroiditis [1]. This seminal discovery prompted Doniach and Roitt to become the first to detect thyroglobulin antibodies in the sera of patients with Hashimoto’s thyroiditis (HT), the cause of which they had been trying to elucidate [2].

Rose made many other key observations in thyroid autoimmunity, including the first understanding of the importance of both major histocompatibility complex (MHC) and non-MHC genes in determining susceptibility, the crucial additional role of environmental factors, and the description of autoreactive T cells in normal, healthy individuals, implying the need for active suppressive mechanisms to prevent autoimmunity arising. He died last year, widely celebrated for his achievements across the now broad field of autoimmunity. This review to honour his passing will focus on developments in our understanding of autoimmune thyroiditis since 2015, the year of a previous update [3], hopefully giving a snapshot of where we have got to at this juncture. As before it focuses on predisposing factors and then pathogenic mechanisms, and concludes with a section on disease associations that may provide an insight into those mechanisms, as well as possibly contributing to disease presentation. The term HT is used generically rather than as its original description of lymphadenoid goitre.

## Genetic susceptibility

Although we know something about the heritability of thyroid autoantibodies, it is only recently that accurate estimates have been established for HT. In a large Swedish twin study, the probandwise concordance rate for HT was 0.29 and 0.1 for monozygotic and dizygotic twins respectively, giving an estimated heritability of 0.64 [4]. This was less than for type 1 diabetes mellitus (0.81) and Addison’s disease (0.97) in the same population, emphasising the importance of both genetic and environmental factors in determining susceptibility. Familial co-aggregation of the other autoimmune diseases with HT was less common than might have been expected but the higher concordance in monozygotic twins confirmed that such disease sharing was dependent on common genes.

Most attempts to look at the genes which predispose to autoimmune thyroid disease have recently focussed on Graves’ disease rather than HT but an attempt has been to look at how much the established or tentative polymorphisms associated with HT actually contribute. In a relatively small series of 142 Polish cases, only seven polymorphisms could be confirmed as being associated, contributing 5.5% of the overall variability, and none of the usual environmental factors appeared to be associated with susceptibility either, presumably due to sample size [5]. Another study from Croatia of 405 patients, with a confirmation cohort of a further 303, identified three novel variants contributing 4.8% of the genetic variance in HT, but due to limited power the authors could not confirm associations already established by conventional association studies [6].

Much larger studies are clearly needed but these will be difficult to organise and fund, given the perception that HT is less important than Graves’ and other diseases. At this stage, we can see that there must be dozens of genes at least, each contributing a tiny fraction to the overall complex picture. Vitiligo serves as a good comparator; it has a similar heritability and prevalence to HT. Approximately 60 common genetic variants account for over two-thirds of total vitiligo heritability, and these have effect sizes greater than other complex diseases, meaning that genome-wide screening has been a greater success in vitiligo than in HT and other autoimmune disorders [7]. It has also been possible to impute that there is no ‘missing heritability’ (for instance due to epistasis or epigenetic effects) in vitiligo, whereas this remains an assumption for most other autoimmune diseases. Nonetheless identifying the myriad rare and private genetic variants that account for 29% of the genetic risk in vitiligo will take huge populations or a fresh approach; this challenge will be far greater still in HT where rare variants seem to predominate.

One recent example of a likely private gene contributing to HT is the description of a previously unreported splice site variant in the thyroglobulin gene (*TG* c. 1076-1G > C), associated with exon skipping, and resulting in a variant transcript of *TG*; this variant was found in affected members of a family with apparently autosomal dominant HT, as well as one unaffected child (who may yet develop HT or represent incomplete penetrance) [8]. It is already known that certain *TG* polymorphisms are associated with HT as well as Graves’ disease, but confer very modest risk. Whether the novel variant encodes a thyroglobulin molecule that injures thyroid cells, leading to autoimmunity, or provokes an immune response through alternate mechanisms is not yet known but it is noteworthy that affected members had autoantibodies to both thyroglobulin and thyroid peroxidase (TPO). Another family with autosomal dominant inheritance of HT has been reported with a mutation leading to haploinsufficiency of the gene encoding tumour necrosis factor-α-induced protein 3, also known as A20 [9]. It is already known that A20 haploinsufficiency may result in inflammatory and autoimmune disorders, presumably through the role of A20 in regulating T helper 17 (Th17) cells and other immune responses, and another such patient with HT in association with Behçet’s syndrome and vitiligo has been reported recently [10]. Consanguinity has been associated with an increased relative risk of 3.3 for HT in a study of 298 Jordanian HT patients when compared to healthy controls [11]. Such unions lead to increased expression of autosomal recessive but not autosomal dominant or X-linked disorders, and further studies of such examples may be fruitful.

More conventional candidate gene association studies have continued to add potential candidates to those already identified (Table 1) but are often of small size and have not yet been replicated. A potentially important example is the tumour necrosis factor superfamily member 4 gene which encodes OX40 ligand (CD252), a co-stimulatory signal expressed by many antigen-presenting cells. Polymorphisms in this gene have been associated with autoimmune rheumatological conditions and a weak association has now been reported with HT in young Han Chinese patients [12]. The same group, possibly using overlapping patient cohorts, has also looked at five ubiquitin gene polymorphisms, failing to find any association with Graves’ disease but reporting one weak unconfirmed association with HT [13]. To my mind these studies demonstrate some of the pitfalls associated with such an approach: failure to include a replication cohort and to correct for the number of variables tested, compounded by initially grouping discrete disorders together as ‘autoimmune thyroid disease’ and then separating them out for post hoc analysis.

**Table 1:** Summary of the key genetic, environmental, and existential factors associated with Hashimoto’s thyroiditis

*Genetic factors*
Major histocompatibility genes (HLA class I and II)
Immunoregulatory genes (e.g. *CTLA4, PD1, CD40*)
Thyroid-specific genes (*TG*)
Genes associated with thyroid peroxidase antibody synthesis (e.g. *TPO, BACH2*)
*Environmental factors*
Iodine intake—possible U-shaped relationship
Smoking—protective but increased risk when stopped
Alcohol—protective
Selenium—evidence equivocal
Vitamin D—evidence equivocal
Infections—evidence equivocal except for congenital rubella and hepatitis C; may operate through childhood exposure (the hygiene hypothesis) or gut microbiota
Therapies which modulate the immune system; lithium; irradiation
*Existential factors*
Female sex
Pregnancy
Age
Down’s syndrome
Associated disease—prolactinoma, allergic rhinitis, subacute thyroiditis (all equivocal)

## Environmental factors

An increasing numbers of environmental factors are known to affect susceptibility to HT and are summarised in Table 1. Most attention over the last 5 years has focussed on selenium, especially as a possible therapeutic agent, the gut microbiota and drugs. Data on both the association of selenium intake with markers of autoimmune thyroiditis and the effects of selenium supplementation on such markers have been conflicting but despite this, a survey of 881 members of the Italian Associazione Medici Endocrinologi found that almost 80% prescribed selenium for HT associated with euthyroidism, despite around half of the respondents not actually believing that the evidence supported its use [14]. About two-thirds considered using selenium in subclinical hypothyroidism in association with TPO antibodies, presumably with the aim of trying to slow disease progression. A meta-analysis has demonstrated that selenium supplementation in HT patients treated with levothyroxine reduced serum TPO antibody levels after 3, 6, and 12 months, but a reduction was seen only at 3 months and not at 6 and 12 months in untreated HT patients [15]. Thyroglobulin antibody levels fell less reliably still and overall evidence quality was deemed low. Even these equivocal effects on antibody levels cannot be taken as indicating any actual clinical benefit, especially considering the increase in reported adverse effects. Future results, such as the outcome of the CATALYST trial, will be required before the use of selenium can be generally endorsed [16].

Another area of uncertainty has been the possible association between month of birth and risk of HT. A recent large and rigorous twin cohort study from Denmark indicated a 2% increased risk for developing HT in women born in the summer as opposed to other seasons [17]. The reason for this effect is not known but could reflect exposure to some infectious agent, vitamin D levels or another unknown factor. Maintaining healthy gut microbiota is important in gastrointestinal homeostasis and metabolism but also affects the development and maturation of the lymphoid system, best shown by the immunological changes seen in germ-free animals. There is increasing evidence for a role of altered gut microbiota in a range of diseases, including autoimmunity, and two recent reports have found alterations in gut microbiota in HT. In one study this involved patients who were hypothyroid, thus complicating the picture due to the possible effects of thyroid hormone deficiency on gut function [18], whereas the other study comprised euthyroid HT patients [19]. Clearly much needs to be done to understand whether these changes have any aetiological significance but a role for microbiota would fit with old observations in experimental autoimmune thyroiditis, in which animals raised in specific-pathogen-free conditions had lower levels of thyroiditis and thyroglobulin antibodies [20]. It is noteworthy in the context of HT seasonality that the offspring of specific-pathogen-free animals treated in late pregnancy with oral antibiotics and conventional intestinal contents were similarly more susceptible to autoimmune thyroiditis than the offspring of non-treated counterparts.

Drugs that modulate the immune system may cause HT, the best known example being α-interferon (IFN). Recent studies have shown that thyroid cells exposed to α-IFN in vitro have increased levels of thyroglobulin mRNA but reduced protein levels due to lysosomal degradation, which could lead to the release of pathogenic thyroglobulin peptides in vivo [21]. As other autoimmune diseases also arise after α-IFN treatment, there must be additional pathways which ultimately result in such complications. Immune checkpoint inhibitors (ICIs) are therapeutic monoclonal antibodies which inhibit the CTLA-4 or programmed death-1 (PD1) pathways: ipilimumab is a CTLA-4 inhibitor, nivolumab and pembrolizumab inhibit PD1 and atezolizumab, avelumab and duvalumab inhibit PD ligand 1. ICIs are aimed at removing the brakes on T cell activation, thus enhancing T cell responses against tumour cells that are otherwise suppressed, but since such suppression is also involved in self-tolerance, they have the unwanted side effect of causing autoimmune disorders [22]. Thyroid dysfunction occurs in 5–10% of ICI-treated patients, being greatest with anti-PD agents, especially when given in combination with CTLA-4 inhibition [23, 24]. It presents most commonly as hypothyroidism, often preceded by destructive thyroiditis which seems to be mediated by cytotoxic T cells; there is intra-thyroidal predominance of CD8^+^ and CD4^−^CD8^−^ T lymphocytes [25]. The detection of thyroid autoantibodies has been inconsistent and has no obvious predictive role, unlike the situation with α-IFN [26]. It is also now clear that alemtuzumab, which is used in multiple sclerosis to deplete T cells and frequently leads to Graves’ disease during the phase of immune reconstitution, may cause HT in around 15% of cases, as well as painless thyroiditis [27]. The preferential occurrence of Graves’ disease may reflect the association of this type of immune reconstitution with other autoantibody-mediated autoimmune diseases. It will fascinating to unravel how much these new induced forms of thyroiditis can tell us about the pathogenesis of HT and painless thyroiditis.

## Pathogenetic mechanisms

The relationship between goitrous and atrophic forms of autoimmune thyroiditis has long been debated, particularly the question of whether the former simply progresses to the latter, as opposed to these being two discrete entities. IgG4-related disease (IgG4-RD) is a recently recognized disorder affecting a range of tissues and characterised by the infiltration of IgG4-positive plasma cells, stromal fibrosis and elevated serum IgG4 concentrations. Around 30% of patients with HT in Japan and the United States have IgG4-RD affecting the thyroid and this is associated with early-onset hypothyroidism and thyroid atrophy, but the prevalence in Europe appears to be much lower at 12% [28]. It is not clear whether this difference relates to methodological issues, the relative iodine deficiency of the particular European population studied or other factors, but it does favour the concept that thyroid atrophy in HT is usually the result of disease progression.

Regulatory T cell (Treg) are now well established as having a critical role in preventing autoimmunity. Several types have been identified, broadly classed as natural or thymic Tregs (CD4^+^ CD25^+^Foxp3^+^), previously shown to be abnormal in HT, and induced Treg subsets which differentiate in the periphery in response to specific antigen. An increase in CD4^+^CD69^+^Foxp^−^ Tregs but with diminished function has been reported in HT [29], and a decrease in both number and function of CD4^+^CD49^+^LAG-3^+^IL-10^+^ Treg type 1 cells has also been found [30]. These changes have been identified using peripheral blood samples and also occur in Graves’ disease. It seems that the relationship between Treg and HT is complex by the time disease is fully established, but future detailed studies, especially using thyroid-derived lymphocytes, could identify the sequence of immunoregulatory failure that leads to autoimmune thyroid disease.

In terms of thyroid cell injury, cytokines derived from the lymphocytic infiltrate play a key role, including their ability to stimulate the thyroid cells themselves to release proinflammatory mediators, thus amplifying and perpetuating the autoimmune response [3]. Previous studies have shown that blood and thyroid Th17 cells which secrete the cytokine IL-17 are increased in HT, as in many other autoimmune disorders, but a recent study has reported finding an additional source of IL-17 in the thyroid follicular cells themselves in HT [31]. Expression correlated with the appearance of CD68^+^ macrophages inside the follicle although there was no evidence to support a direct role of IL-17 in tight junction dysfunction. In addition to IL-17, Th17 cells secrete IL-22, a cytokine which targets epithelial cells and which is also secreted by Th22 cells. High levels of Th22 cells have now been reported in the blood and thyroid of HT patients [32] and determining the effect of IL-22 on thyroid cells will be of interest. IL-21 is a pleiotropic cytokine which plays a key role in the development of Th17 cells. Elevated levels of this cytokine have been detected in the thyroid and blood of HT patients and thyroid follicular cells also show greater IL-21 receptor expression in HT than in Graves’ disease or controls [33]. A further proinflammatory cascade has been identified in HT with the finding of increased expression of multiple inflammasome components (NLRP1, NLRP3, NLRC4, AIM2, ASC, and caspase-1) and their associated cytokines (IL-18 and IL-1β) in the thyroid of patients, as well as identification of inflammasome component release by thyroid cell stimulated in vitro with the IFN-γ and tumour necrosis factor-α, which may in turn contribute to further cytokine release and cell death through pyroptosis [34].

The PD-1/PD ligand-1 axis has also been investigated. This pathway plays a major role in suppressing adaptive immunity in a variety of settings, including the immune response by tumour cells, and as we have seen, blockade can result in autoimmune disease. PD ligand-1 is expressed by thyroid follicular cells in both HT and Graves’ disease in areas of the gland associated with the presence of PD-1^+^ T cells, and IFN-γ is capable of inducing PD ligand-1 expression in primary thyroid cell cultures and lines [35]. This raises the possibility that such expression could help to maintain peripheral tolerance when there is ongoing inflammation, akin to the way that HLA class II expression by thyroid cells operates in the absence of a suitable co-stimulatory signal [36], and it may also explain the appearance of destructive thyroiditis after ICI treatment.

## Thyroid autoantibodies

A recent study of largely euthyroid patients with thyroid nodules found that the presence of TPO antibodies or diffuse heterogeneity on ultrasound examination showed high specificity (89.4% and 88.9%, respectively) but only moderate sensitivity (63.9% and 49.1%, respectively) in identifying histologically-defined coincidental HT [37]. The positive predictive value of TPO antibodies for the presence of HT was only 75%, reminding us that in the evolution of HT, circulating autoantibodies are not essential for disease initiation. It is also important to recall that the diagnostic accuracy of thyroid autoantibodies is more dependent on the type of assay used than is usually acknowledged. In a survey using 5 different assay kits, there was the discordance between positive and negative results for thyroglobulin and TPO antibodies, and correlation between kits ranged widely [38]. For 4 of the assays, thyroglobulin antibodies were more frequent (99%) that TPO antibodies (81%) in HT, making the former the test of choice in this Japanese cohort.

Thyroid autoantibodies have been found to associate with a variety of symptoms such as depression and impaired quality of life independently of thyroid hormone levels, presumably reflecting an adverse effect of the ongoing autoimmune process on health and wellbeing. Two recent studies have added to this evidence. In the first, thyroglobulin and TPO antibody levels showed a negative correlation with quality of life scores in HT patients but there was no correlation between autoantibody levels and thyroid function tests [39]. In the second, both autoantibodies showed a correlation with a symptom score comprising 16 symptoms of hypothyroidism, but again no correlation was found with thyroid hormone levels; thyroglobulin antibodies were associated with particular symptoms in a logistical regression model [40].

Perhaps the most persuasive evidence that the autoimmune process contributes to symptom burden in HT comes from a study of hypothyroid HT patients who continued to experience symptoms after euthyroidism was restored with conventional replacement treatment. Total thyroidectomy followed by thyroxine replacement was associated with an increase in general health score, a decrease in fatigue score and a decrease in chronic fatigue frequency from 82 to 35%, whereas the medically treated control group had no improvement [41]. Thyroidectomy was associated with a marked fall in TPO antibody levels but there are several pathways by which autoimmunity could theoretically produce such effects besides one which is antibody-mediated, most obviously via cytokines. The duration of follow-up was only 18 months, the patient group was not representative of the whole HT population and of course it was not a true controlled trial as sham-thyroidectomy was not carried, but the results are a striking proof-of-principle that suppressing the autoimmune process in HT may have benefit in some patients.

Thyroid-stimulating hormone receptor (TSHR) antibodies detected by the standard binding inhibition assays occur in a proportion of HT patients but more complex bioassays are require to determine their functional significance. A recent large cross-sectional survey identified TSHR-blocking antibodies in 9.3% of HT patients but only around a half were hypothyroid; while some of the euthyroid group appear to have been taking thyroxine, others had TSHR-stimulating antibodies in addition, which may have countered the effect of TSHR blockade [42]. In another study by the same group, 6% of 700 patients with HT were found to have clinical evidence of thyroid-associated ophthalmopathy and TSHR-stimulating antibodies were present in 69% of these cases, in contrast to only 6% of HT patients without eye signs [43]. Patients with more active and severe ophthalmopathy had higher TSHR-stimulating antibody levels but despite this two-thirds of the patients with ophthalmopathy were hypothyroid, presumably on the basis of cell-mediated cytotoxicity.

## Associations with HT

Disease associations with HT may shed further light on pathogenesis. A comprehensive study of over 25,000 offspring of HT patients has confirmed familial associations among 20 of 43 other autoimmune disorders, 6 of which (autoimmune haemolytic anaemia, chronic rheumatic heart disease, chronic glomerulonephritis, immune thrombocytopenic purpura, pemphigus and Takayasu disease) were not found in a similar cohort of Graves’ patients [44]. In addition modest spouse correlation was found within the overall familial risk, indicating some sharing of environmental risk factors, but the overwhelming risk was genetic and shared with other autoimmune disorders. Such disease clustering is more common in adults with HT than in children and shows a different pattern, with arthropathies and connective tissue diseases being more frequent in HT in adults and type 1 diabetes mellitus (T1DM) and coeliac disease more frequent in children and adolescents with HT [45]. Skin diseases showed no age-related pattern, with vitiligo being the most common such associated disorder. The association of HT with T1DM has been examined at a molecular level with the description of an HLA class II peptide-binding pocket (DRβ-Tyr26, DRβ-Leu67, DRβ-Gln70, DRβ-Lys71 and DRβ-Arg74) that is strongly associated with this particular disease cluster, and demonstration that the pocket can bind peptides derived from thyroglobulin, TPO and glutamic acid decarboxylase 65 (a key autoantigen in T1DM) and cause T cell activation [46].

Of possible relevance to the question of unresolved symptoms in HT discussed in the last section, a systematic review of the association between rheumatological conditions and HT confirmed the well-recognised excess of inflammatory arthritis, but also found evidence for an association with osteoarthritis, fibromyalgia and chronic widespread pain [47]. Evidence quality was too low to perform a meta-analysis. Another intriguing association is the finding of HT in 16% of a series of 1239 patients with allergic rhinitis whereas Graves’ disease was not found [48]. Allergic rhinitis depends on a Th2 response while HT has been assumed to reflect more of a Th1 response, so these results add further weight to the increasing understanding that a Th1/Th2 dichotomy is far less clear in man than in the mouse. A role for prolactin in modulating autoimmune responses has been suggested previously by animal and human studies, although this is not always acknowledged. A prospective study of prolactinoma patients has found a three-fold excess of HT compared to controls, in line with the results of a recent retrospective survey [49, 50].

Perhaps the most perplexing disease relationship with HT has been its apparent association with the rare development of steroid-responsive encephalopathy, with some arguing that these patients merely represent a coincidental overlap between a common autoimmune disorder and a broader group of steroid-responsive encephalopathies. The situation is not helped by the variability in presenting features, ranging from mild cognitive impairment to status epilepticus, meaning that the diagnosis may well be overlooked. Although the presence of thyroid autoantibodies is diagnostic the pathogenesis is unclear, but thyroid hormone status plays no obvious role. A recent systematic survey has identified only 251 cases of steroid-responsive encephalopathy associated with HT in the literature; electroencephalography was abnormal in 82% and there was a suggestion that those patients with thyroglobulin antibodies but without TPO antibodies have a better prognosis [51]. Thyroid autoantibodies were present in the cerebrospinal fluid in 76% of patients but this alone cannot be taken as evidence of any aetiological link. It seems more likely that a cross-reactive autoantigen present in brain and thyroid could be involved, and protein disulphide-isomerase A3 has been investigated as one such candidate. Mice immunised with protein disulphide-isomerase A3 develop an elevated TSH and impaired learning and memory, as well as antibody-mediated complement fixation in both thyroid and brain tissue [52].

More work in all these areas is needed to confirm the associations, identify any causal relationship and explore the immunological implications, but it seems obvious that HT is a more complex disorder than often appreciated. Much research still remains to be done 70 years on from Noel Rose’s first discoveries.
